# Catechin prevents the calcium oxalate monohydrate induced renal calcium crystallization in NRK-52E cells and the ethylene glycol induced renal stone formation in rat

**DOI:** 10.1186/1472-6882-13-228

**Published:** 2013-09-17

**Authors:** Wei Zhai, Junhua Zheng, Xudong Yao, Bo Peng, Min Liu, Jianhua Huang, Guangchun Wang, Yunfei Xu

**Affiliations:** 1 Department of Urology, Shanghai Tenth People’s Hospital, Tongji University School of Medicine, Shanghai 200072, China

**Keywords:** Renal tubular cell injury, Renal calcium crystallization, Catechin, In vivo, In vitro

## Abstract

**Background:**

Reactive oxygen species play important roles in renal calcium crystallization. In this study, we examined the effects of catechin, which have been shown to have antioxidant properties on the renal calcium crystallization.

**Methods:**

In the vitro experiment, the changes of the mitochondrial membrane potential, expression of superoxide dismutase (SOD), 4-hydroxynonenal (4-HNE), cytochrome c, and cleaved caspase 3 were measured to show the effects of catechin treatment on the NRK-52E cells induced by calcium oxalate monohydrate (COM). In the vivo study, Sprague–Dawley rats were administered 1% ethylene glycol (EG) to generate a rat kidney stone model and then treated with catechin (2.5 and 10 mg/kg/day) for 14 days. The urine and serum variables were dected on 7 and 14 days after EG administration. The expression of cytochrome c, cleaved caspase 3, SOD, osteopontin (OPN), malondialdehyde (MDA), 8-hydroxy-2′-deoxyguanosine (8-OHdG) in kidney were measured. Furthermore, the mitochondrial microstructure in the kidney was also examined by transmission electron microscopy.

**Results:**

Catechin treatment could prevent the changes in mitochondrial membrane potential and expression of SOD, 4-HNE, cytochrome c, and cleaved caspase 3 in NRK-52E cells induced by the COM. For the in vivo experiments, the EG administration induced renal calcium crystallization was also prevented by the catechin. The expression of SOD, OPN, MDA, OPN and 8-OHdG, were increased after EG administration and this increase was diminished by catechin. Moreover, catechin also prevented EG induced mitochondrial collapse in rat.

**Conclusions:**

Catechin has preventive effects on renal calcium crystallization both in vivo and in vitro, and provide a potential therapeutic treatment for this disease.

## Background

Nephrolithiasis is the condition marked by the development of renal stones. Renal stones are aggregates of crystals that are formed in supersaturated urine [1]. Calcium oxalate may occur as multiple stones or may recur, can induce pain with both passage and obstruction, and is commonly caused by treatable metabolic disorders of hypercalciuria [2]. It appears as a complex disease that may develop change-able clinical manifestations [3]. Despite the increasing prevalence of the disease in China, little research is available for indicating the mechanism and prophylaxis for renal calculus in detail.

It has already known that exposure to high levels of oxalate and calcium oxalate crystals can induce oxidative stress such as an increase in free radical generation, increased lipid peroxidation, a decrease in cellular anti-oxidant status and an increase in phospholipase-A2 (PLA2)-induced release of arachidonic acid [4-6]. Sustained exposure to high levels of oxalate or calcium oxalate crystals injures the cells [7]. Mitochondria have been demonstrated to show excessive uptake of calcium when the cytoplasm level of free calcium markedly increases, causing abnormalities in the respiratory chain and increasing the mitochondrial production of ROS (Reactive Oxygen Species) [8,9]. Calcium-induced mitochondrial injury can be prevented by antioxidants suggesting that oxidative stress may be an important event in its development [8].

Renal tubular cell injury is induced by the oxidative stress, which is produced during the attachment of crystals to renal tubular cells [10]. Exposure to high concentrations of oxalate can give rise to the generation of ROS, mitochondrial collapse and increased lipid peroxidation, which induces the cell death in cultured renal epithelial cells [11,12]. Renal toxicity is assumed to be caused by the elevation of serum-free iron concentration, following its reduction at the luminal side of the proximal tubule, which generates ROS decreasing antioxidant systems and also leads to the enhancement in lipid peroxidation [13,14]. It has been demonstrate that toxic action of acephate on kidney cells is partly through an ROS-mediated mechanism [15]. And ROS are known to mediate many toxin induced renal tubular injuries [16-18].

In renal tubular cell injury, mitochondrial damage has been recognized as a crucial cause for tubular cell death which involves disruption of respiration complexes and loss of mitochondrial membrane potential [19,20]. And cell apoptosis is precipitated by mitochondrial outer membrane permeabilization and consequent release of apoptogenic factors such as cytochrome c [21]. Caspases are crucial mediators of programmed cell death (apoptosis). Among them, caspase-3 is a frequently activated death protease, catalyzing the specific cleavage of many key cellular proteins [22]. Caspase-3 is activated in the apoptotic cell both by extrinsic (death ligand) and intrinsic (mitochondrial) pathways [23,24]. In intrinsic activation, cytochrome c from the mitochondria works in combination with caspase-9, apoptosis-activating factor 1 (Apaf-1), and ATP to process procaspase-3 [22,25,26].

Catechin, regarded as one of the main components of green tea, is considered to exert antioxidant effects acting directly as radical scavengers or metal-chelators and also indirectly through modulation of transcription factors or enzymes [27,28]. As ROS plays an important role in renal calcium crystallization, catechin may have the therapeutic effect.

Therefore, we administered catechins to renal proximal tubular cell and rats with stone formation to investigate its inhibitory effects on urolithiasis. In this study, we examined the changes in oxidative stress in an in vitro study and in vivo study. We further showed that renal calcium crystallization was significantly decreased by catechin treatment. Together, these results provide compelling evidence for a role of oxidative stress, which activates the initial process of renal calcium crystallization and can be prevented by catechin.

## Methods

### Preparation of calcium oxalate monohydrate (COM) crystal suspensions

Oxalic acid (200 mM, 0.5 ml) and 200 mM calcium chloride we remixed at room temperature to a final concentration of 10 mM, and the COM crystals that immediately formed in suspension were equilibrated for 3 days. The COM crystals were then washed three times with sodium and chloride-free distilled water saturated with calcium oxalate, resuspended to a final concentration of 2.92 mg/ml, and adjusted to pH 6.8.

### Cell culture

The renal proximal tubular cell line NRK-52E (the Type Culture Collection of the Chinese Academy of Sciences, Shanghai, China.) was cultured in Dulbecco’s modified Eagle’s medium (GIBCO, USA) and was supplemented with 10% newborn calf serum,100 IU/ml penicillin, and 100 μg/ml streptomycin (Life Technologies, Burlingont, ONT, Canada). The cells were routinely seeded at a density of 1 × 10^6^/60-mm culture dish (Corning Incorporated, Glendale, Arizona, United States) at 37°C in a humidified atmosphere of 5% CO_2_ in air. The medium was changed every three day and the cells were subcultured before forming confluent monolayers.

NRK-52E cells were seeded at a density of 1 × 10^6^/90-mm dish and cultured to 90% confluence. The cells were then treated with or with-out catechin hydrate (Beyotime Biotechnology, Haimen, Jiangsu, China) (0.4 μl/ml) for 10 min and then with COM crystals (80 μg/cm^2^). In our study, cell suspension was prepared and counted in the blood counting chamber.

### Measurement of mitochondrial membrane potential (Δψm)

Cells were loaded with the membrane potential-sensitive dye tetramethylrhodamine ethyl ester perchlorate (TMRE; 20 nM in Hepes-buffered salt solution; Invitrogen, Carlsbad, CA, USA). Cells loaded with TMRE were then analyzed using a confocal micro-scope (LSM5 PASCAL; Carl Zeiss Co. Ltd., Oberkochen, Germany) equipped with × 20 and × 100 oil-immersion, quartz objective lenses. The cells were then treated with or without CsA (Cyclosporin A, 2 μM) for 10 min and then with COM crystals (100 μg/cm2) for 0, 5, 10, 15, and 30 min. As a negative control, untreated NRK-52E cells were ob-served, and as a positive control, we used carbonyl cyanide m-chlorophenyl hydrazone (CCCP; 10 μM), an uncoupler that causes mitochondrial depolarization. Mitochondrial fidelity in cells stained with TMRE was quantified by flow cytometry. After three washes with phosphate-buffered saline (PBS) to remove COM crystals, stained cells in each group were detached using 0.05% trypsin – EDTA (Ethylene Diamine Tetraacetic Acid), washed with PBS, and diluted to 1 ml. A total of 30,000 events were collected from each sample and the data were displayed on a logarithmic scale of increasing red fluorescence intensity using a FACS Calibur HG (Becton–Dickinson, Franklin Lakes, NJ, USA).

### Experimental animals

All experimental procedures were performed with the approval of the Animal Care Committee of the Faculty of Medicine, Tongji University. Male Sprague–Dawley (SD) rats (Charles River Japan, Yokohama, Japan), age 7 weeks weighing approximately 280 g were used. Animals were provided with a standard mEq diet (containing calcium, 1.12 g; phosphorus, 0.9 g; magnesium, 0.26 g; and sodium, 0.21 g/100 g; Oriental Yeast Co., Tokyo, Japan) and free access to water. To induce calcium oxalate deposition in the kidneys rats were given 2 doses of 0.12 ml 5% ethylene glycol (EG) (Wako, Tokyo, Japan) and then treated twice per day with catechin through a stomach tube. The rats were assigned to one of the following groups (n = 10 per group) and weighed weekly: one group received EG only (EG group) and two EG and catechin groups also received 2.5 and 10 mg/kg/day catechin (EG + catechin 2.5, EG + catechin 10.0 groups, respectively). At 7 and 14 days after the start of drug therapy, blood was sampled from the inferior vena cave of 5 rats per group. These rats were sacrificed under ether anesthesia and both kidneys were immediately excised and dissected. One kidney was histologically examined and RNA was extracted from the other. Two days before euthanasia, the rats were placed individually in metabolic cages for 24 h and urine samples were collected into cups containing HCl for oxalate measurements. The kidneys and urine samples were obtained from control rats without EG or catechin at day 0 (n = 5).

### Western blotting

NRK-52E cells stored at - 20°C were immersed in 1 × lysis buffer and lysed by sonication on ice. The total protein concentration in the supernatant was spectrophotometrically quantified using an Ultrospec 3100 Pro (GE Healthcare, Wallingford, CT, USA). Samples containing 30 μg total protein were mixed with loading buffer (Laemmli sample buffer; Bio-Rad Laboratories, Hercules, CA, USA).

Proteins were resolved by 12.5% sodium dodecyl sulfate–polyacryl-amide gel electrophoresis and transferred onto Immobilonpolyviny-lidene fluoride membranes (Millipore, Bedford, MA, USA). Blocking for 1 h at room temperature was followed by an overnight incubation with one of the following antibody: anti-rat malondialdehyde (MDA) antibody, anti-rat 8-hydroxy-2′-deoxyguanosine (8-OHdG) antibody, polyclonal anti-rat superoxide dismutase (SOD) antibody (dilution 1:100; Santa Cruz Biotechnology, Santa Cruz, CA, USA), anti-rat 4-hydroxy-2-nonenal (4-HNE) monoclonal antibody (dilution 1:4; Nikken Seil, Shizuoka, Japan), and anti-rat cleaved caspase 3 antibody (dilution 1:1000; Cell Signaling Technology, Beverly, MA, USA) at 4°C. After being washed, the membranes were treated with the corresponding peroxidase-conjugated secondary antibodies for 1 h at room temperature. Proteins were visualized using enhanced chemiluminescence Western blotting analysis kits (PierceBiotechnology). The membranes were probed with a β-actin anti-body as a loading control (Sigma – Aldrich, St. Louis, MO, USA). For Western blotting of cytochrome c, a similar procedure was followed except for the use of samples (cytosol fraction isolated from NRK-52E cells) containing 10 μg total protein and anti-rat cytochrome c antibody (dilution 1:1000; Cell Signaling Technology).

The same membrane was used for catechin (-) COM (+) and catechin (+) COM (+) group to ensure uniformity. The protein expression levels in the bands corresponding to SOD, 4-HNE, cytochrome c, and cleaved caspase 3 (n = 5 each) were quantified using Image Quant LAS 4000 (GE Healthcare Japan, Tokyo, Japan), which is a multipurpose CCD (Charge Coupled Device) camera system for quantitative imaging of blots developed by Amersham for enhanced chemiluminescence, with standard UV (ultraviolet) transillumination for ethidium bromide gel visualization.

### Measurement of urinary and serum variables

Urinary volume and urinary pH were measured manually. Urinary calcium and serum creatinine, serum calciumwere determined using an automated analyzer (Model 705, Hitachi, Tokyo, Japan). Urinary oxalate was determined using oxalate decarboxylase and citrate was determined by citrate lyase conversions to oxaloacetate.

### Transmission electron microscopy (TEM)

The microstructure of mitochondria in the kidney was examined using TEM. Kidneys were perfusion- fixed using 20 ml of 0.1 M phosphoric acid buffer and 20 ml of 2.5% glutaraldehyde, extracted and washed with phosphoric acid buffer, and then fixed with 2% osmium tetraoxide for 2 h. Tissues were dehydrated using a graded series of ethanol (50– 100%), embedded in epoxy resin, and polymerized at 60°C for 48 h. The ultrathin sections (99 nm) were double stained with uranium and lead for observation using a JEM-1011 TEM microscope (JEOL Ltd., Tokyo, Japan).

### Statistical analysis

All data are expressed as means ± standard deviation. The statistical significance of differences among groups was examined using the Mann–Whitney U test. A P value of < 0.05 denotes a statistically significant difference.

## Results

### Changes in mitochondrial membrane potential in NRK-52E cells exposed to COM or COM&catechin

**Figure 1:**
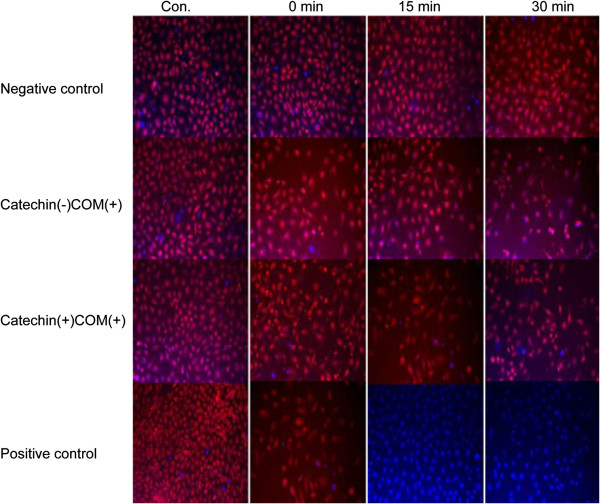
**NRK-52E cells stained with TMRE (laser confocal fluorescence microscopy × 40).** Dots aggregated in mitochondria with red fluorescent bodies were positive staining, while blue fluorescent ones showed mitochondria around nuclei.

NRK-52E cells stained with TMRE, which are aggregated in mitochondria with red fluorescent bodies, while blue fluorescent ones show mitochondria around nuclei (Figure 1). The negative control cells indicated no change in TMRE intensity from 0 min to 30 min. The TMRE intensity of catechin (-) COM (+) cells exposed to COM crystals gradually decreased during 0 min to 30 min. On the other hand, red fluorescent bodies in catechin (+) COM (+) cells exposed to both catechin and COM crystals had no significant change from 0 min to 30 min. In the positive control, TMRE intensity completely disappeared within 15 min and 30 min.

**Figure 2:**
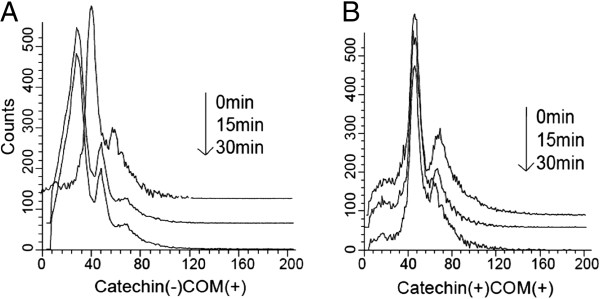
**The results of flow cytometry of NRK-52E cells stained with TMRE.** The COM treated NRK-52E cells without catechin administration is shown as **(A)**. NRK-52E cells treated with both COM and catechin were shown as **(B)**. The assays were carried out at 0,15 and 30 min.

Form the results of flow cytometry, the peak TMRE intensity of catechin (-) COM (+) cells was shifted to the left at 15 min and shifted slightly more to the left at 30 min, compared to that of the catechin (-) COM (+) cells at 0 min (Figure 2A). However, in catechin (+) COM (+) cells, the shift in peak TMRE intensity was negligible during 0 min to 30 min (Figure 2B).

### Expression changes of SOD, 4-HNE, cytochrome c and cleaved caspase 3 in NRK-52E cells exposed to COM or COM&catechin

**Figure 3:**
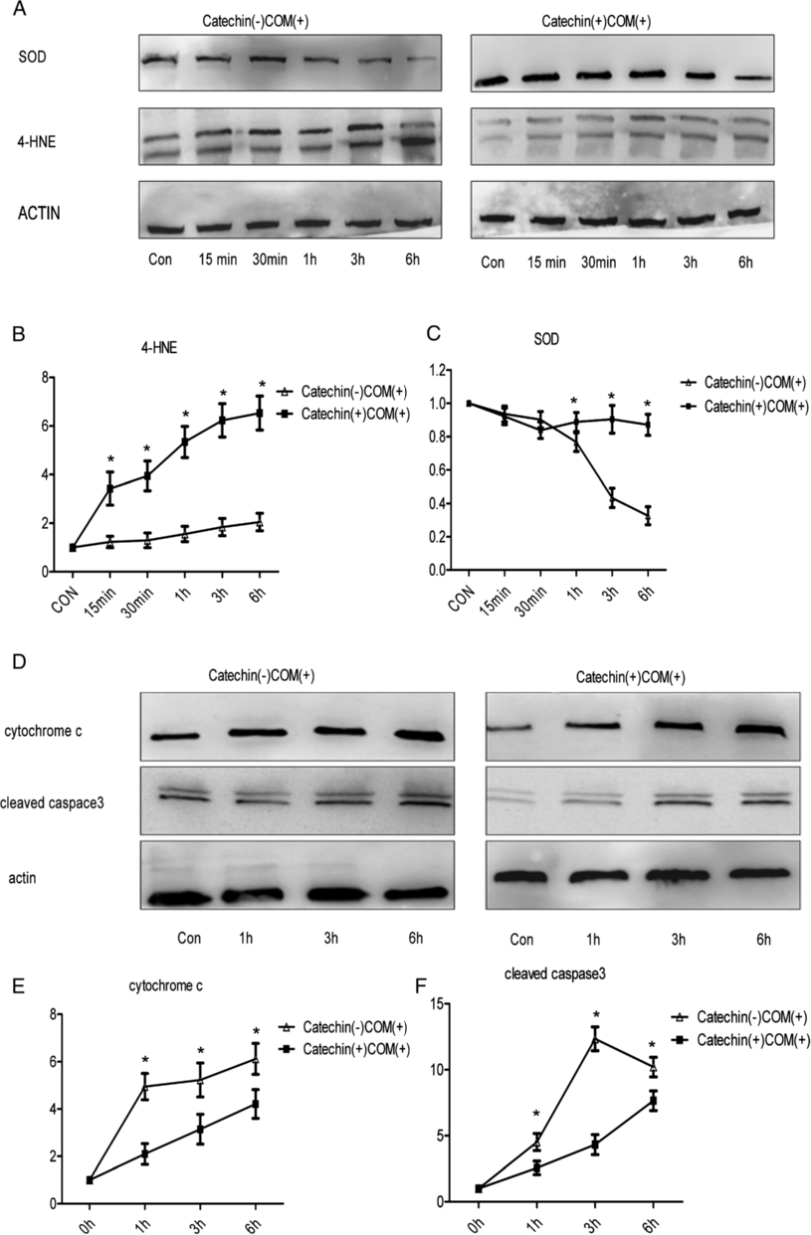
**The expression of superoxide dismutase (SOD), 4-hydroxynonenal (4-HNE), cytochromec, and cleaved caspase 3.** Proteins were subjected to Western blotting using antibodies against SOD, 4-HNE **(A)** cytochrome c, and cleaved caspase 3 **(D)** and quantified by densitometric scanning, results being expressed in arbitrary units. Figure **(B)** and **(C)** present the expression of SOD, 4-HNE, while **(E)** and **(F)** for cytochrome c, and cleaved caspase 3. Values are the mean ± SEM for five independent determinations. *Statistically significant difference (P < 0.05), catechin (-) COM (+) vs. catechin (+) COM (+).

In catechin (-) COM (+) cells, SOD expression decreased gradually in a time-dependent manner, whereas no significant changes were detected in catechin (+) COM (+) cells (Figure 3A). There were obviously differences in the expression of SOD between catechin (-) COM (+) cells and catechin (+) COM (+) cells at 1, 3, and 6 h after exposure to COM crystals (P < 0.05, Figure 3C).

Meanwhile, the expression of 4-HNE increased gradually from 0 min to 6 h in catechin (-) COM (+) cells, however, it was a gentle increase in catechin (+) COM (+) cells (Figure 3A). The change of 4-HNE expression between catechin (-) COM (+) cells and catechin (+) COM (+) cells was significantly observed at 15 min, 30 min, 1 h, 3 h and 6 h after exposure to COM crystals (P < 0.05, Figure 3B).

For the expression of cytochrome c, in catechin (+) COM (+) cells, the expression increased at 1 h, 3 h, and 6 h after exposure to COM crystals, but the expression was lower than that in catechin (-) COM (+) cells (Figure 3D). The change of cytochrome c expression between catechin (-) COM (+) cells and catechin (+) COM (+) cells was significantly observed at 1 h, 3 h, and 6 h after exposure to COM crystals (P < 0.05, Figure 3E).

The expression of cleaved caspase 3 was increased from 0 h to 6 h in catechin (+) COM (+) cells. The expression of cleaved caspase 3 increased from 0 h to 3 h whereas reduced from 3 h to 6 h in catechin (-) COM (+) cells (Figure 3D). There were significant differences in the expression of cleaved caspase 3 between catechin (-) COM (+) cells and catechin (+) COM (+) cells at 1 h, 3 h and 6 h after exposure to COM crystals (P < 0.05, Figure 3F).

### Urinary and serum variables in the control and crystal-model rat

There were no changes in urine volume or urinary pH on 7 and 14 days after EG administration. In the EG group, urine volume was significantly lower than in the EG + catechin10.0 group on 14 days. In the EG group, 24-hour urine oxalate excretion was significantly higher than in the control group after administration (P < 0.05). The 24-hour urine oxalate of EG + catechin2.5 group was more than 5.6-fold and 8.7-fold higher than that of the control group on 7 and 14 days, respectively. The 24-hour urine oxalate of EG + catechin10.0 group was more than 6.4-fold and 8.2-fold higher than that of the control group on 7 and 14 days, respectively (P < 0.05, Table 1).

**Table 1:** The results of the measurement of urinary and serum variables

**Group (day)**	**Urine volume (ml)**	**Urinary PH**	**Urine (mg/day)**		**Serum (mg/dl)**	
			**Oxalate**	**Calcium**	**Creatinine**	**Calcium**
Control						
7	17.54 ± 3.7	8.36 ± 0.3	1.88 ± 0.3	3.4 ± 1.09	0.22 ± 0.05	10.83 ± 0.47
14	17.04 ± 4.9	8.49 ± 0.7	1.77 ± 0.5	3.45 ± 2.46	0.23 ± 0.08	11.29 ± 0.85
EG						
7	16.54 ± 6.6	8.44 ± 1.3	13.57 ± 1.5*	4.13 ± 0.87	0.28 ± 0.12	11.52 ± 0.23
14	16.05 ± 7.5	8.85 ± 0.5	13.58 ± 1.3*	3.87 ± 1.12	0.36 ± 0.13	10.50 ± 0.13
EG + catechin2.5						
7	21.28 ± 1.7	8.76 ± 0.7	10.55 ± 1.1*	3.23 ± 0.45	0.26 ± 0.09	11.06 ± 0.45
14	22.01 ± 4.8	8.55 ± 2.1	15.46 ± 0.9*	3.73 ± 0.68	0.27 ± 0.05	11.01 ± 0.43
EG + catechin10.0						
7	19.32 ± 2.4	8.56 ± 0.7	12.05 ± 1.3*	4.65 ± 1.32	0.20 ± 0.04	11.07 ± 0.36
14	27.05 ± 7.5	8.77 ± 1.4	14.53 ± 2.4*	4.14 ± 1.78	0.23 ± 0.06	11.21 ± 0.43

The statistical significance of differences among groups was examined using the Mann–Whitney U test. *, P < 0.05.

On the other hand, there were no significant differences in 24-hour urine calcium among the control group, the EG group and the EG + catechin groups. Meanwhile, there were no marked differences in serum creatinine and calcium levels among the control, EG, and EG + catechin groups on 7 and 14 days after administration (P > 0.05, Table 1).

### Immunohistochemical staining for SOD, osteopontin, MDA and 8-OHdG in the control and crystal-model rat kidneys

**Figure 4:**
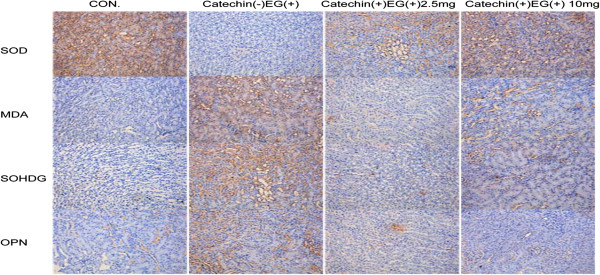
**Th immunohistochemical staining of superoxide dismutase (SOD), malondialdehyde (MDA), 8-hydroxy-2′-deoxyguanosine (8-OHdG), and osteopontin (OPN) in the kidney of the rat on day 14.** All the images represent serial sections. EG, EG + catechin2.5, EG + catechin10.0 represent rats administered with EG or EG and catechin at 0, 2.5, and 10.0 mg/kg/day, respectively. Strong expression of SOD was could be observed in the control group, but SOD was not detected in EG group. The expression of SOD was slightly lower in EG + catechin 2.5 group than that in EG + catechin10.0 group. OPN was barely detectable in the control group, but it was strongly expressed in the EG group. The OPN expression in EG + catechin2.5 group was slightly higher than that in EG + catechin10.0 group. MDA was undetectable in the control group and EG + catechin groups, but it was detectable in EG group kidneys. The expression of 8-OHdG was undetectable in the control group, but it was detectable in the EG group. And the expression of 8-OHdG was nearly undetectable in the EG + catechin groups.

Strong expression of SOD could be observed in the control group, but SOD was not detected in EG group. The expression of SOD was slightly lower in EG + catechin 2.5 group than that in EG + catechin10.0 group (Figure 4).

Osteopontin (OPN) was also tested by immunohistochemical staining. OPN was barely detectable in the control group, but it was densely distributed throughout the renal tubular cells of whole kidneys in the EG group. OPN expression was slightly higher in EG + catechin2.5 group than in EG + catechin10.0 group (Figure 4).

MDA was undetectable in the renal tubular cells of whole kidneys in the control group, but it was detectable in EG group kidneys. In the EG + catechin groups, MDA production was undetectable (Figure 4).

Similarly, the expression of 8-OHdG was undetectable in the renal tubular cells of whole kidneys in the control group, but it was detectable in the nuclei of renal tubular cells of whole kidneys in the EG group. In the EG + catechin groups, the expression of 8-OHdG was nearly undetectable (Figure 4).

**Figure 5:**
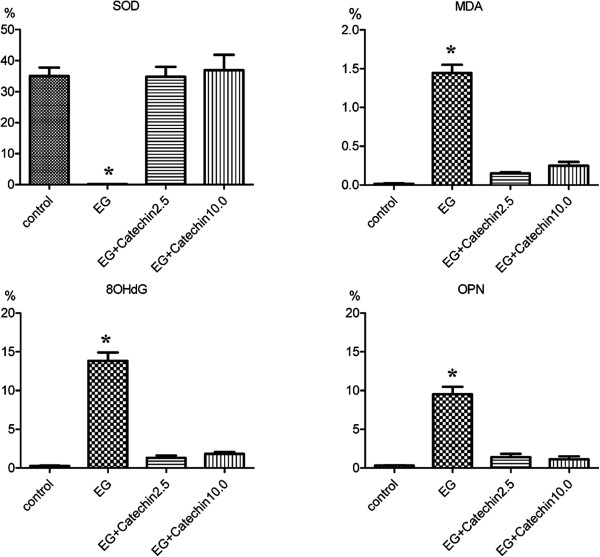
**The results of the semi-quantification of immunohistochemical staining of SOD, OPN, MDA and 8-OHdG in the kidney of the rat on day 14.** EG, EG + catechin2.5, EG + catechin10.0 represent rats administered with EG or EG and catechin at 0, 2.5, and 10.0 mg/kg/day, respectively. *, P < 0.05.

Finally, the ratios of the OPN, MDA and 8-OHdG expression areas were significantly higher in the EG group than those in the other groups (P < 0.05, Figure 5). Meanwhile, there was no significant difference in the ratios of SOD, OPN, MDA and 8-OhdG expression areas among the control group, EG + catechin 2.5 group and EG + catechin 10.0 group (P > 0.05, Figure 5).

### OPN, cytochrome c and cleaved caspase 3 expression changes evaluated by western bolting in the control and crystal-model rat

**Figure 6:**
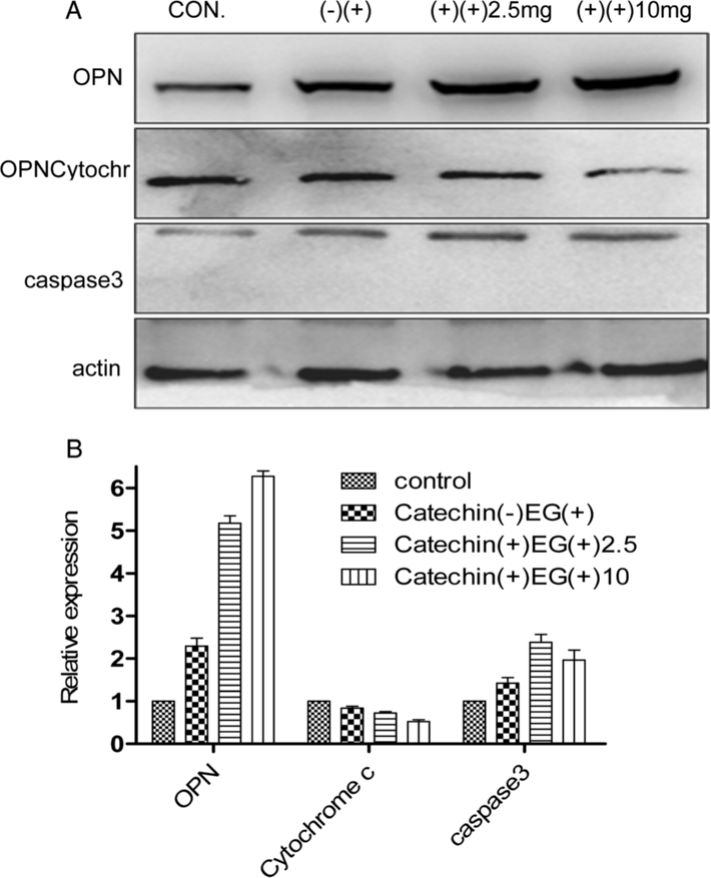
**The expression changes of OPN, cytochrome c and cleaved caspase 3 were detected by western bolting on day 14 (A).** The relative expression values of OPN, cytochrome c and cleaved caspase 3 were compared **(B)**. EG, EG + catechin2.5, EG + catechin10.0 represent rats administered with EG or EG and catechin at 0, 2.5, and 10.0 mg/kg/day, respectively.

OPN expression increased gradually after EG treatment in rat. In EG + catechin 2.5 group and EG + catechin10.0 group, the expression of OPN was higher than that in EG group (P < 0.05). Additionally, administration of catechin after EG treatment enhanced the expression of OPN. There was a significant change in OPN expression between the control and EG group. A significant differences between EG group and EG + catechin groups were also observed (P < 0.05, Figure 6).

The expression of cytochrome c had no changes among the four groups. There was a significant change in cleaved caspase 3 between the control and EG + catechin 2.5 group (P < 0.05). There were no significant differences among the other three groups for the expression of cleaved caspase 3 in crystal-model rat kidneys (P > 0.05, Figure 6).

### Ultrastructural findings of kidneys exposed to EG in the control and crystal-model rat

**Figure 7:**
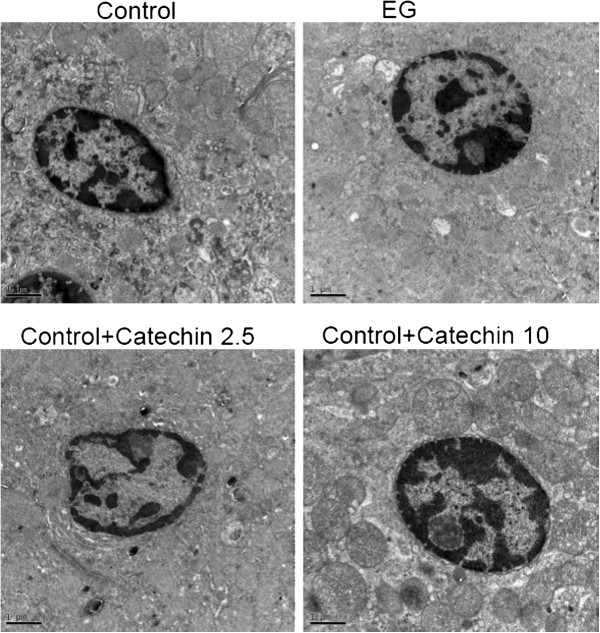
**The ultrastructure of rat kidney sections examined by TEM on 14 days.** The renal tubules were circular, microvilli were evident in the lumen, and mitochondria were located around the nuclei in the control group. In the EG group, the renal tubules were thin with flattened tubular cells; the lumen of the renal tubule was expanded; microvilli were barely recognizable; crystals were present in the lumen; swollen mitochondria resembling fat droplets around the nuclei had an indistinct, discontinuous, and partly collapsed double membrane. In the EG + catechin (EG + catechin 2.5, and EG + catechin 10.0) groups, the renal tubules were circular and microvilli were detected in the lumen; the renal tubules were longer than those in the EG group, and the layer was thicker, but slightly shorter than that in the control group; the mitochondria had a regular internal structure with a continuous double membrane which was similar to the mitochondria in the control group. Scale bars, 1 μm.

The renal tubules were circular, microvilli were evident in the lumen, and mitochondria were located around the nuclei in the control group (Figure 7).

In contrast, renal tubules of the EG group were thin with flattened tubular cells, the lumen of the renal tubule was expanded, microvilli were barely recognizable, and crystals were present in the lumen (Figure 7). Swollen mitochondria resembling fat droplets around the nuclei had an indistinct, discontinuous, and partly collapsed double membrane. However, the renal tubules of the EG + catechin (EG + catechin 2.5, and EG + catechin 10.0) groups were circular and microvilli were detected in the lumen; they were longer than those in the EG group, and the layer was thicker, but slightly shorter than that in the control group. The mitochondria had a regular internal structure with a continuous double membrane, similar to the mitochondria in the control group.

## Discussion

Tea catechins, a subclass of compounds in the flavonoid family, have been found to have several biologically beneficial properties including antioxidative effects [29,30]. Flavonoids leading to cytoprotective effects against oxidative stress. Our findings, taken together with other observations [27,30-32], indicate that catechin demonstrates renoprotective abilities in several models of renal disease, especially nephrolithic nephropathy. The experiments indicate that catechins can attenuate functional and immunohistochemical changes in the renal proximal tubular cell line NRK-52E treated with COM and kidneys of EG induced nephrolithic rats.

In our in vitro study, catechin attenuated the changes of mitochondrial membrane potential, and normalized expression of SOD, 4-HNE, cytochrome c, and cleaved caspase 3 in the renal proximal tubular cell line NRK-52E treated with COM.

The disappearance of TMRE in NRK-52E cells indicated mitochondrial collapse through depolarization of the mitochondrial membrane. Disappearance of TMRE was gradual after exposure to COM crystals. COM crystals stimulate renal tubular cells to generate superoxide (O2•-) via NADPH oxidase [33]. Furthermore, O2• - leads to mPTP opening, which is considered to induce mitochondrial collapse [34]. The opening of the mPTP changes the Δψm, which can be monitored using TMRE, a fluorescent probe that accumulates in polarized mitochondria and is released upon their depolarization. As catechin is a scavenger of free radicals or reactive oxygen species [35], it may attenuate the superoxide released by NADPH oxidase, and leads to normalize potential of the mitochondrial membrane.

Decreased superoxide dismutase and increased 4-hydroxynonenal are often used as indexes of oxidative stress and cell injury. The generation of reactive oxygen species have been found to increase after the COM treatment in other studies [1,36]. The increase of anti-oxidant enzymes (SOD) and lipid peroxidation products (4-HNE) is likely a result of higher level of oxidative stress [37]. Binding of the released cytochrome c to cytosolic apoptosis protease activating factor 1 activates caspase 9 and caspase 3, which results in the induction of apoptosis. During this process, ROS are released from the intramembrane compartment into the cytosol, which further injures renal tubular cells. In our study, the increased expression of cytosolic cytochrome c and cleaved caspase 3 indicated that mitochondrial collapse and cell apoptosis could be induced by COM crystals.

In the in vivo study, oxidative stress was evaluated by SOD, MDA, and 8-OHdG expression in rats with EG induced renal calcium crystallization, which prevented by catechin. Oxalate and calcium oxalate (CaOx) crystals are injurious to renal epithelial cells [38]. For monitoring DNA damage, 8-OHdG are the general marker [39], whereas MDA is examined for lipid peroxidation [40]. Our results showed that, mitochondria in the EG + catechin groups showed a regular internal structure with a continuous double membrane similar to that of mitochondria in the control group, while mitochondria in EG group showed an indistinct, discontinuous, and partly collapsed double membrane. This indicated that catechin prevented mitochondrial collapse, which induced by EG.

In the in rats with EG induced renal calcium crystallization, similar to the observation in NRK-52E cells, we also found the increase of cytochrome c release and the activation of caspase 3, which leads to the apoptosis or necrosis. These lipids disrupt mitochondrial function by increasing ROS, decreasing mitochondrial membrane potential, and increasing mitochondrial permeability [41]. Moreover, calcium oxalate (CaOx) kidney stones are formed attached to Randall’s plaques, which is the subepithelial deposits on renal papillary surfaces and triggered by reactive oxygen species [12].

As ROS plays an important role in the process of the development of the nephrolithiasis, antioxidant therapy has been described. In the previous studies, they found that vitamin E administration has been shown to prevent calcium oxalate precipitation in the rat kidney and decreased urinary oxalate excretion in patients with kidney stones [36]. It also restored antioxidant levels in the blood and decreased the urinary excretion of oxalate and calcium in patients who underwent surgical stone removal [42]. A previous study of antioxidant enzyme levels in rats with stone formation showed that almost all antioxidant enzyme activities were attenuated except that of catalase [43]. In agreement with previous studies, our results confirmed and extended the observation that the oxidative stress was greatly reduced by catechin treatment, which is an antioxidant component of green tea.

The expression of OPN was evenly and densely distributed throughout renal tubular cells of whole kidneys in the EG group. While in the control group and in the EG + catechin groups, the expression of OPN was barely detectable or localized to limited renal tubular cells. OPN, which identified as a major component of stone matrix protein, its expression was remarkably increased in the renal tubular cells of stone-forming rats [44,45]. Some studies suggested that OPN is an inhibitor of abnormal calcification in rat kidneys [46,47]. However, other studies suggested that OPN plays a role in stimulating the deposition and adhesion of crystals to cells in the early stages of crystallization. It is showed that calcium oxalate monohydrate crystal coating with OPN is correlated with increased adhesion tendency [48,49]. It is also reported that the kidney crystal deposition is decreased in OPN-deficient mice compared to crystal deposition in wild-type mice [50].

## Conclusions

In conclusion, the results of our study have showed that catechin have preventive effects on renal calcium crystallization both in vivo and in vitro. However, the specific molecular mechanisms of catechin in renal calcium crystallization need to be further studied.

## Competing interests

The authors declare that they have no competing of interests.

## Authors’ contributions

J-HZ mainly contributed to the preparation of the manuscript and carried out the study described above. WZ, ML, Y-FX collected the data, and Y-FX reviewed the manuscript. All authors read and approved the final manuscript.

## Pre-publication history

The pre-publication history for this paper can be accessed here:

http://www.biomedcentral.com/1472-6882/13/228/prepub
